# Non-Coding Transcript Heterogeneity in Mesothelioma: Insights from Asbestos-Exposed Mice

**DOI:** 10.3390/ijms19041163

**Published:** 2018-04-11

**Authors:** Emanuela Felley-Bosco, Hubert Rehrauer

**Affiliations:** 1 Laboratory of Molecular Oncology, Lungen- und Thoraxonkologie Zentrum, University Hospital Zurich, Sternwartstrasse 14, 8091 Zürich, Switzerland; 2 Functional Genomics Center Zurich, ETH Zurich and University of Zurich, 8057 Zurich, Switzerland; hubert.rehrauer@fgcz.ethz.ch

**Keywords:** mesothelioma heterogeneity, non-coding RNA, long-non-coding RNA

## Abstract

Mesothelioma is an aggressive, rapidly fatal cancer and a better understanding of its molecular heterogeneity may help with making more efficient therapeutic strategies. Non-coding RNAs represent a larger part of the transcriptome but their contribution to diseases is not fully understood yet. We used recently obtained RNA-seq data from asbestos-exposed mice and performed data mining of publicly available datasets in order to evaluate how non-coding RNA contribute to mesothelioma heterogeneity. Nine non-coding RNAs are specifically elevated in mesothelioma tumors and contribute to human mesothelioma heterogeneity. Because some of them have known oncogenic properties, this study supports the concept of non-coding RNAs as cancer progenitor genes.

## 1. Introduction

Protein coding genes make up only 2% of the human genome. In the remaining part of the genome, many transcriptionally active regions are found that give rise to non-coding RNA (ncRNA) [1]. Long non-coding RNAs (lncRNAs) are defined as longer than 200 nucleotides and represent the major class of ncRNAs since there are nearly three times as many lncRNA genes as protein-coding genes [2,3], and recently there has been a steep increase in research focusing on lncRNAs owing to their impact in several biological processes [4,5]. The class of non-coding RNAs that are smaller than 200 nucleotide includes the microRNA (miRNA, 19–25 nucleotides) that post-transcriptionally regulate gene expression via the suppression of specific target mRNAs [6].

LncRNA expression plays a crucial role in regulating the gene expression during differentiation and development [7,8]. For a few lncRNAs, functional characterization is available and indicates an association with transcriptional regulation and post-transcriptional processing of coding regions. Specifically, these lncRNAs affect miRNA expression, mRNA stability, and translation [9]. One of the first lncRNAs described to contribute to cancer was the HOX antisense intergenic RNA (*HOTAIR*)—this lncRNA interacts with chromatin and represses the transcription of human HOX genes, thus regulating development [10]. Several lncRNAs have been identified to be involved in the various hallmarks of cancer causing various tumor types including lung, liver, prostate, breast, and ovarian cancers [11,12,13].

Mesothelioma is a rare, aggressive cancer developing from the mesothelium and it is mostly associated with exposure to asbestos [14]. Recent molecular analyses have defined four different types of mesothelioma on the basis of gene expression [15], and two molecularly defined groups associated with different prognosis [16]. In this study, we explore the variation of non-coding RNA expression associated with this heterogeneity. In order to prioritize which ncRNA might be the most relevant in a given cancer type, it has been suggested that by using the The Cancer Genome Atlas (TCGA) ncRNAome information as a clinical filter, one would be able to generate a reduced and clinically relevant ncRNA list that could be used for a candidate-oriented functional screening. Here, we take the opportunity of our recent study in asbestos-exposed C57Bl/6J *Nf2*^+/−^ mice [17], to identify lncRNAs and miRNAs associated with tumor development and scrutinize their expression and heterogeneity in human mesothelioma and human mesothelioma TCGA RNAome. *Nf2* heterozygote background was chosen based on the fact that *NF2* mutations are often observed in mesothelioma [18,19,20,21], and a previous study showing its contribution to tumor development [22]. 

## 2. Results

**Figure 1:**
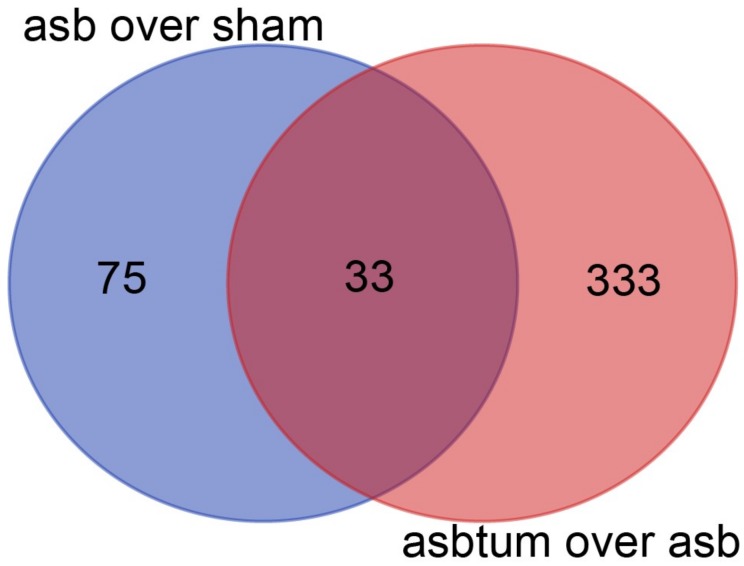
Overlap of the differentially expressed non-coding genes (more than 2-fold change, *p* < 0.01) in crocidolite-exposed vs. sham (asb over sham) and crocidolite-exposed with tumors vs. crocidolite-exposed (asbtum over asb) comparisons visualized as a Venn diagram.

We analyzed the expression of non-protein-coding RNA in the RNA-seq data [17] obtained in tissue extracted from either C57Bl/6J *Nf2*^+/−^ mice that were exposed eight times to crocidolite (blue asbestos) every three weeks, or sham-treated mice. Mice had been sacrificed 33 weeks after first crocidolite exposure in order to have the possibility of investigating pre-cancer and cancer stages. In order to identify gene expression changes during mesotheliomagenesis, we have analyzed three treatment groups by RNA-seq: sham, age-matched crocidolite-exposed, and age-matched crocidolite-exposed with observable tumors. We performed differential expression analysis between crocidolite-exposed and sham, and identified 108 non-protein-coding genes with more than 2-fold expression (*p* < 0.01, False Discovery Rate (FDR) < 0.017). Differential expression analysis between crocidolite-exposed with tumors and crocidolite-exposed, identified 366 non-protein-coding genes with more than 2-fold expression (*p* < 0.01, FDR < 0.024). 33 genes were found in both comparisons, as shown in the Venn diagram (Figure 1). 

We selected some of them based on (a) the significance of their differential expression in tumor vs. crocidolite-exposed inflamed mesothelium and (b) the availability of some functional knowledge about them (Table 1). 

Then we compared the selected ncRNAs to differentially expressed genes with more than two-fold increased expression between inflamed tissue from crocidolite and sham (Table 1, last column). Of the 14 selected genes, three (*Dios3os*, *Dubr*, and *Morrbid*) were also overexpressed in inflamed crocidolite-exposed tissues compared to tissues from sham-treated mice.

**Figure 2:**
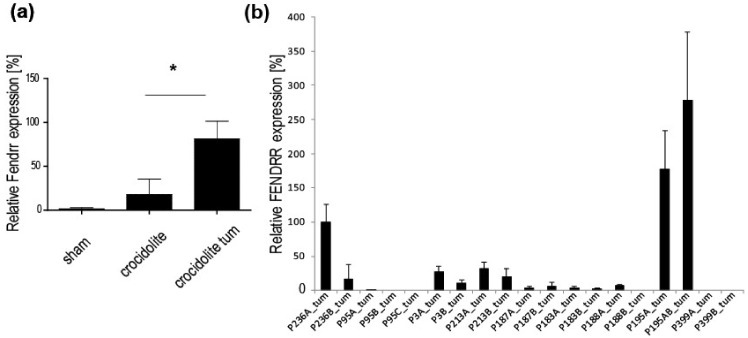
Fendrr is overexpressed in mice mesothelioma and associates with epithelial histotype commitment in human mesothelioma. (**a**) q-PCR of *Fendrr* expression was performed in sham, crocidolite-exposed mice without malignant tumors. Mean ± SE, *N* = 5–8 mice. * *p* < 0.05, Mann–Whitney test. (**b**) FENDRR gene expression analysis in tumor samples from nine patients for whom tissue is available at different time points during the progression of the disease. Mean ± SD, *N* = 3.

The ncRNA gene with the highest upregulation in mesothelioma tumor in mice exposed to asbestos was *Fendrr* (*Fetal-lethal noncoding developmental regulatory RNA*) and we validated this finding by quantitative-PCR (Figure 2a). We then took the opportunity to investigate its expression in tumor tissue collected at different time (Figure 2b) during tumor progression in nine patients. We have recently deeply characterized genomic alterations in two out of these nine patients [23]. Interestingly, *FENDRR* expression was increased in the tissue of the patient, which had maintained epithelioid histology (P236A_tum and P236B_tum), compared to the patient that had initially been diagnosed as epitheloid mesothelioma (P95A_tum) but where we have observed epithelial to mesenchymal transition (EMT) during tumor progression. We could detect FENDRR expression in all first tumor samples from patients diagnosed with epithelioid mesothelioma but not in patient P399, who had been diagnosed with biphasic histology.

**Figure 3:**
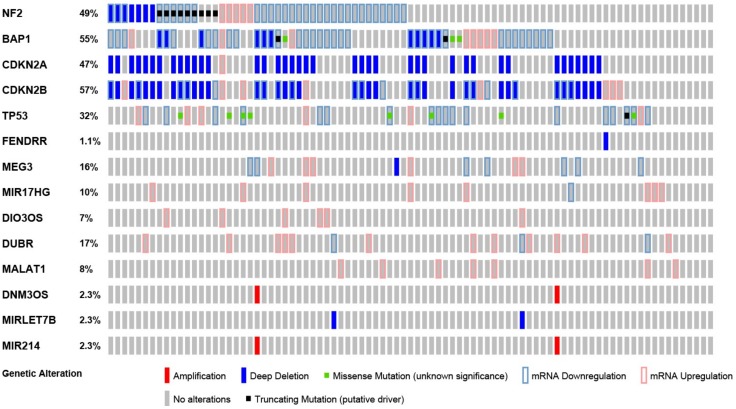
Non-coding RNAs contribute to mesothelioma heterogeneity. “Oncoprint” analysis performed using cBioportal of selected ncRNAs and five tumor suppressor genes frequently mutated in mesothelioma.

The existence of known orthologs in human for 13 of the selected ncRNAs allowed us to evaluate their contribution to tumor heterogeneity by interrogating publicly available TCGA data of 87 MPM samples (MESO) through the cBioPortal [24,25] together with five tumor suppressor genes frequently mutated in mesothelioma (Figure 3). For *HOXA-AS2* (*lncRNA–HOXA cluster antisense RNA 2*), *FIRRE* (*functional intergenic repeating RNA element*) and *MORBIDD* (*myeloid RNA regulator of Bim-induced death*), no differences were detected in TCGA data; therefore, they were not included in the figure. All other ncRNAs contribute to tumor heterogeneity.

Interestingly *DNM3OS* (*dynamin 3 (Dnm3) gene antisense*) is amplified in two patients and consistent with *DNM3OS* being a precursor for *miR214*, the latter is amplified as well. Although DNM3OS overexpression is associated with enrichment is sarcomatoid histotype compared to epithelioid histotype in Bueno et al [15], in TCGA samples it is amplified in a patient bearing a biphasic and a patient bearing an epithelioid tumor.

In human mesothelioma miRlet7b was deleted in a patient bearing a biphasic and a patient bearing an epithelioid tumor, indicating that it possibly contributes to epithelial heterogeneity. *FENDRR* was deleted in a patient with biphasic histotype, which would fit with the observation that it is enriched in epithelioid mesothelioma, but this observation is based only on a single patient.

Interestingly there is a significant co-occurrence of alterations of *BAP1* and *DIO3OS* (*p* = 0.024), and of *DUBR* and *miRlet7b* (*p* = 0.028).

## 3. Discussion

In order to improve the treatment of mesothelioma, it is necessary to better understand how molecular heterogeneity contributes to tumor growth.

We report here the likely contribution of ncRNAs to the heterogeneity profile and suggest that oncogenic driver events in mesothelioma development are associated with lncRNA expression. This extends the current view that focuses on the loss of tumor suppressor functions as drivers.

*Fendrr* is transcribed divergently from the transcription factor-coding gene *Foxf*1. *Fendrr*-deficiency results in mice lethality due to lack of proper differentiation of mesenchymal derived tissue [26,27]. This lincRNA is predominantly nuclear and physically associates with the PRC2 Polycomb complex [28]). In humans the orthologous transcript is expressed from a syntenic region [29]. Silencing *FENDRR* increases FN1 expression in gastric cancer cells and increases their migration [30]. Interestingly, *FENDRR* is among the genes enriched in the epithelioid compared to sarcomatoid mesothelioma cluster based on gene-expression profile [15].

Not much is known about *gm26902*, except that its expression characterizes a subset of microglia CD11c+ population, which sustains brain development [31], while expression of *gm17501* has been associated with cardiac hypertrophy [32]. 

Meg3 (maternally expressed 3) binds to p53 and activates the transcription of a part of p53-regulated genes [33]. In gastric cancer, MEG3 increases Bcl-2 levels by sequestering miR-181-a [34]. In addition, MEG3 modulates the activity of TGF-β pathway genes by binding to distal regulatory elements, which have GA-rich sequences, allowing MEG3 specific binding to the chromatin through RNA–DNA triplex formation [35,36]. 

miR17-92 cluster (*miR-17-92a-1 cluster host gene*) binds HuR, a member of the ELAVL family, which has been reported to contribute to the stabilization of AU-rich elements (ARE)-containing mRNAs, possibly modulating HuR activity on target mRNA stability [37]. MiR 17-92 cluster is amplified in high-grade B-cell lymphoma with Burkitt lymphoma signature, resulting in higher expression of miR17-92 and lower expression of *BIM* and *PTEN* and increased BCR signaling [38]. It is noteworthy that miR17-92 expression is increased in mesothelioma [39].

Dio3os is transcribed in the antisense orientation to Dio3, which codes for the type 3 deiodinase, an enzyme-inactivating thyroid hormones that is highly expressed during pregnancy and development [40].

*Dubr* (also called Dum: developmental pluripotency-associated 2 (Dppa2) Upstream binding Muscle lncRNA) silences its neighboring gene, Dppa2, in *cis* through the recruitment of Dnmt1, Dnmt3a and Dnmt3b, thereby promoting myoblast differentiation and damage-induced muscle regeneration [41].

Malat1 (Metastasis-associated lung adenocarcinoma transcript 1) expression results in alternatively spliced transcripts [42]. It is for example necessary for correct splicing of B-Myb, a transcription factor involved in G2/M transition [43]. In patients with early-stage non-small cell lung cancer high levels of MALAT1 predict a high risk of metastatic progression [44]. *Malat1* loss of function in mouse revealed that it is a nonessential gene in development or for adult normal tissue homeostasis [45,46], but depletion of *MALAT1* in lung carcinoma cells impairs cellular motility in vitro and metastasis in mice [47]. Therefore, it has been suggested that *MALAT1* overexpression in cancer may drive gain-of-function phenotypes not observed during normal tissue development or homeostasis. Its action seems mediated not only by regulation of alternative splicing, as mentioned above, but also possibly through interaction with HuR [48] like for miR17-92 cluster. 

*Firre*-encoded lncRNA serves as a platform for trans-chromosomal association by interacting with the nuclear matrix factor heterogeneous nuclear ribonucleoproteins U (hnRNPU) through a 156-bp repeating sequence and localizes across a ~5-Mb domain on the X chromosome [49]. It was suggested that it modulates nuclear architecture across chromosomes [49]. Transcription of *FIRRE* is regulated by NF-κB signaling in macrophages and intestinal epithelial cells [50]. Indeed, FIRRE positively regulates the expression of several inflammatory genes in macrophages or intestinal epithelial cells in response to lipopolysaccharide stimulation via posttranscriptional mechanisms including interaction with hnRNPU, which controls the stability of mRNAs of selected inflammatory genes through targeting the adenine-rich element of their mRNAs [50].

Dnm3os is essential for skeletal muscle formation and body growth during development and it serves as precursor of miR214 [51,52]. It is enriched in the sarcomatoid mesothelioma subtype cluster compared to epithelioid [15].

Hoxaas2 directly interacts with enhancer of zeste homolog 2 (EZH2) and lysine-specific demethylase 1 (LSD1), promoting pancreatic cell growth [53].

*Morrbid* is highly and distinctively expressed by mature eosinophils, neutrophils, and classical monocytes in both mice and humans [54]. Interestingly it could be a marker of exposure to carcinogenic fibers since it is overexpressed in tissues of mice exposed to long carcinogenic compared to short non-carcinogenic asbestos and also long compared to short nanotubes [55].

miRlet7 downregulates interferon β (IFNβ) and is upregulated in macrophages upon IFNβ treatment [56].

Although the method that we have used to extract RNA was not optimal for miRNA analysis we detected the overexpression of miR214, likely because of Dnm3os overexpression. MiR214 downregulates PTEN [57] and Sufu [58].

Although only Malat1, from the ncRNAs mentioned, is an lncRNA for which a clear genetic link with tumorigenesis has been established [59,60], it is likely that ncRNAs function in mesothelioma as “cancer progenitors genes” [61]. In addition to *MALAT1*, overexpression of *miR17-92 cluster* is likely oncogenic and of potential therapeutic interest because it activates druggable pathways. Similarly, overexpression of miR214 possibly indicates activation of Hedgehog and PI3K signaling.

Although for FENDRR the contribution to heterogeneity is based on the observation that it is enriched in epitheloid histotype and that one patient has a deletion in this gene, the fact that it is overexpressed in tumors and associates with epithelioid commitment in the patients analyzed indicate that further studies should explore the role of this lncRNA in mesothelioma.

Because MEG3 has been found to modulate TGF-β activity and it has an heterogeneous expression, it would be interesting to investigate whether its expression plays a role in the EMT signature that we observed in the mesothelioma tumors developing in asbestos-exposed mice [17] and also if it contributes to mesothelioma’s so-called transitional state [62].

In an era where immunotherapy is also being intensively explored in mesothelioma treatment [63], it might be wise to consider the deletion of miRlet7b as a possible biomarker for response. 

In summary, we were able to identify lncRNAs that are overexpressed in mesothelioma and we found that they contribute to human mesothelioma heterogeneity. We suggest that they may indicate pathways for precision medicine. One limitation of our approach might be the fact that in our experimental model we observed only spindeloid tumors, which is the opposite of what is observed in human mesothelioma, where epithelioid histotypes are the most frequent. 

Appropriate functional experiments need to be carried out and it would make sense to establish consortia to validate our hypotheses. There is a plethora of ncRNA genes whose functions we need to understand better. In addition, very instructive functional studies rely on animal models but modeling lncRNA function in mice might be difficult because lncRNAs are conserved at much lower rates compared to protein-coding genes, and therefore orthologs are more difficult to identify. 

## 4. Materials and Methods

### 4.1. Analysis of RNA-Seq Data from Tissue Samples from Asbestos-Exposed Mice

RNA was extracted and analyzed as described in our previous paper, where we characterized the overall transcriptome profile of the same samples [17]. Assessment of miR expression was not optimal because the Qiagen RNeasy kit was used to extract RNA, which does not preserve very short RNAs.

### 4.2. Relative Gene Expression 

*Fendrr* gene expression was conducted as previously described [17] [64,65,66] using the following primers (5’–3’): human: AGTGCACTGTGTGCTCTTAG and GAGGATCTGTGGTTGGGTATTT mouse GAAACCAGAGAGCTCCGAATAG and CTTCTGGTGGAGTCAGATCAAA. As in previous studies, histone 3 and β-actin were used as normalizer genes for human and murine gene expression, respectively. RNA was extracted from human mesothelioma tumors and cDNA was prepared as we recently described [23].

### 4.3. Analysis of Publicly Available Datasets

To analyze the expression and genetic alterations of selected non-coding RNA together with five tumor suppressor genes frequently mutated in mesothelioma, we obtained the data from TCGA, using www.cbioportal.org. For mRNA differential expression we used a *z* score of 1.2, where the *z*-score is the standard deviation of static levels of transcript expression in a given case compared to the mean transcript expression in diploid tumors.

## Figures and Tables

**Table 1:** Selected non-coding RNA more than 2-fold upregulated in murine mesothelioma compared to inflamed crocidolite-exposed mesothelium.

Gene Name	Type	*p*-Value	FDR	Chromosome Location (GRCm38.p5)	Human Ortholog	Upregulation in Crocidolite vs. Sham
*Fendrr*	Divergent lincRNA, nuclear	1.94 × 10^−15^	1.4 × 10^−14^	Chromosome 8: 121,054,882-121,083,110	yes	no
*Gm26902*	lincRNA	1.16 × 10^−9^	4.91 × 10^−9^	Chromosome 19: 34,474,808-34,481,546	no	no
*Gm17501*	lincRNA	3.84 × 10^−5^	8.33 × 10^−5^	Chromosome 3: 145,650,312-145,677,580	no	no
*Meg3*	lincRNA	7.97 × 10^−5^	0.0001805	Chromosome 12: 109,541,001-109,571,726	yes	no
*miR 17-92 cluster*	lincRNA	7.02 × 10^−10^	3.05 × 10^−9^	Chromosome 14: 115,042,879-115,046,727	yes	no
*Dio3os*	antisense	0.003026	0.005339	Chromosome 12: 110,275,384-110,278,068	yes	yes
*Dubr*	linRNA, nuclear	9.36 × 10^−7^	2.79 × 10^−6^	Chromosome 16: 50,719,294-50,732,773	yes	yes
*Malat1*	antisense, nuclear	6.09 × 10^−7^	1.86 × 10^−6^	Chromosome 19: 5,795,690-5,802,672	yes	no
*Dnm3os*	antisense	2.26 × 10^−16^	1.87 × 10^−15^	Chromosome 1: 162,217,623-162,225,550	yes	no
*Hoxaas2*	antisense	5.73 × 10^−7^	1.76 × 10^−6^	Chromosome 6: 52,165,674-52,169,564	yes	no
*Firre*	lincRNA, nuclear	4.09 × 10^−7^	1.28 × 10^−6^	Chromosome X: 50,555,744-50,635,321	yes	no
*Morrbid*	nuclear	1.18 × 10^−7^	3.92 × 10^−7^	Chromosome 2: 128,178,319-128,502,765	yes	yes
*miRlet7b*	miRNA	0.000884	0.001697	Chromosome 15: 85,707,319-85,707,403	yes	no
*Mir214*	mirRNA	1.08 × 10^−5^	2.78 × 10^−5^	Chromosome 1: 162,223,368-162,223,477	yes	no
